# Photodocumentation in oculoplastic surgery: an up-to-date overview

**DOI:** 10.5935/0004-2749.2021-0318

**Published:** 2025-08-22

**Authors:** Juliana Senna Figueiredo Barbi, Izabela Camargos de Figueirêdo Neves, Patrícia Mitiko Santello Akaishi, Ricardo Morschbacher, Julián Espinosa

**Affiliations:** 1 Centro Oftalmológico de Minas Gerais, Belo Horizonte, MG, Brazil; 2 Department of Ophthalmology, Faculdade de Medicina de Ribeirão Preto, Universidade de São Paulo, Ribeirão Preto, SP, Brazil; 3 Universidade Federal de Ciências Médicas de Porto Alegre, Porto Alegre, RS, Brazil; 4 Sociedad Médica Bolivariana EyeClinic, Valledupar, Cesar, Colombia

Photodocumentation is of cardinal importance in medical practice, particularly in specialties that involve aesthetic procedures, such as oculoplastic surgery. The correct standardization of documentation of facial features includes the basic knowledge of digital photography, manual use of camera settings, and regular and steady lighting conditions^(1)^.

Despite several published articles about photography standardization, many medical publications and presentations continue to fail to satisfy a correct clinical and surgical photodocumentation^(2)^.

Thus, this study aimed to provide a narrative review of the existing literature regarding facial clinical photography and describe a self-developed standardization protocol, guided by the literature review. This protocol can be adapted to any oculoplastic surgeon’s practice, without the need to set up a studio in the office or spend on unnecessary photographic equipment.

This study was divided into two parts. The first part consists of a narrative review of the scientific literature on photodocumentation in facial surgery/cosmetic procedures. We conducted a PubMed database research using the following keywords: “photography”, “photographic”, “facial” and Boolean operators “and” “or” to retrieve the papers. The inclusion criteria were the presence of full description of the following parameters: lens, working distance, background, illumination, camera parameters, such as International Standard Organization (ISO), aperture, shutter speed, and head positioning. The exclusion criteria were the absence of description of standardization.

The search retrieved 87 articles, of which 68 were excluded because they did not provide a minimum description of standardization; thus, 19 articles were included in the analysis. The included articles were compared concerning the following parameters: lens, working distance, background, illumination, camera parameters such as ISO, aperture, shutter speed, and head positioning.

The second part of this study presented a description of a self-developed, guided by the literature review, standardization protocol for facial photographic registration used by one of the authors (Barbi, JSF). The parameters were kept constant for preand post-surgical or cosmetic procedures in the facial and periorbital regions. We reproduced this photograph protocol in 324 patients diagnosed with dermatochalasis (n=162), ectropion (n=10), entropion (n=16), ptosis (n=18), eyelid tumors (n=12), orbital pathologies (n=8), and facial wrinkles/ botulinum toxin treatment (n=98).

This study adhered to the tenets of the Declaration of Helsinki and was approved by Ethics Committee oversight statement number (CAAE): 41056920700005141. All patients provided written informed consent forms, which have been archived on the author’s files.

## Technical specifications

The equipment used was as follows: a digital single lens reflex (DSLR) camera model EOS Rebel T6 (Canon, Inc., Tokyo, Japan) with an Advanced Photo System Type C (APS-C) sensor measuring 22.3 mm x 14.9 mm, EF 100 mm f/2.8 Macro USM Lens (Canon, Inc., Tokyo, Japan), a dedicated Canon Speedlite 430EX III-RT (Canon, Inc., Tokyo, Japan) with white diffuser reflector, and a tripod.

The images were taken in the medical office of one of the authors (Barbi, JSF), whose roof is painted white and the walls light gray.

**Figure 1:**
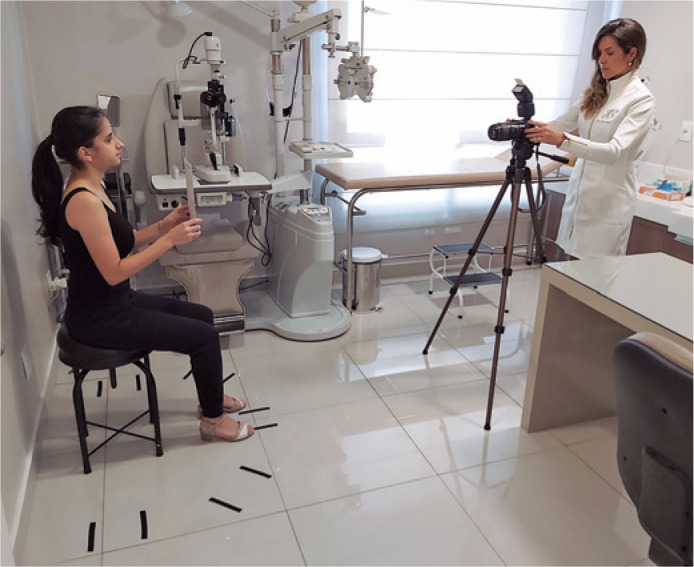
Suggested positioning of the patient and physician, with the patient seated on a swivel stool with no wheels and a floor mat with footmarks for the front, oblique (45°), and profile positions. The camera, preferably mounted on a tripod, must be aligned to the patient’s face with the external flash directed at the ceiling, and a white diffuser reflector attached to the body of the flash was used (white side not visible). The first photo must be taken with the patient holding a 18% gray card so that if any adjustment to the white balance is necessary, the card can serve as a reference in the photo editing software.

For full face and periorbital region composition, the camera was used in manual (M) mode, with f/8 aperture, 1/125 shutter speed, ISO 100, dedicated flash used in the through-the-lens (TTL) automatic mode with 75-degree tilt, and white diffuser reflector. The white balance (WB) was set to “flash” mode, and the focus automatically centered in the patient´s eyes. In two particular situations, settings were modified: in cases of eyelid ptosis, images were taken using the flash from the front, directed toward the patient’s face and diaphragm closing by 1 stop (f/11), and in cases of macro photographs, the flash was directed backward toward a silver diffuser reflector. The patient was seated on a swivel stool without wheels. A floor mat with markings was used to signalize the front (anteroposterior), oblique (45^o^), and profile (90^o^) positions. The camera was positioned on a tripod, and the height was adjusted so that the camera lens was aligned with the patient’s eyes. The external flash was directed at the ceiling and a white diffuser reflector attached to the body of the flash (Figure 1).

**Figure 2:**
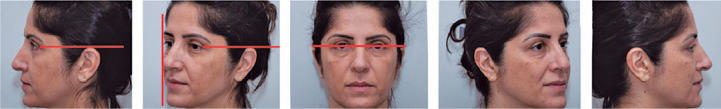
Primary position gaze (PPG). The camera’s thirds grid was used so that the upper horizontal line passes through the pupils and the apex of the patient’s ears. Oblique position (O). The patient is requested to rotate the entire body to 45° until the tip of the nose aligns with the malar eminence and the ear apex with the lateral eyelid canthus. Lateral view framing (L): it is important to align the ear apex to the lateral eyelid canthus and only the same side of Cupid’s bow can be seen.

For full face-framing in the primary position gaze (PPG), the camera’s thirds grid was used so that the upper horizontal line passed through the pupils and the apex of the patient’s ears. For framing the face in the oblique position, the patient was requested to rotate the entire body to 45**°** until the tip of the nose aligned with the malar eminence, and the ear apex aligned with the lateral eyelid canthus. For lateral view framing, it is important to align the ear apex to the lateral eyelid canthus so that only the same side of Cupid’s bow can be seen (Figure 2).

With an APS-C sensor camera and a 100-mm macro lens, the photographer’s was 3 m away from the patient. For periorbital framing, the working distance was 1 m. To ensure and standardize this distance, the patient and photographer were placed on marks on the floor. For macro photographs (e.g., eyelid tumors), the camera was set to manual focus, using a 0.38-m focus distance and 1:1 magnification ratio. In this setting, the photographer had to approach or move away from the lesion to be photographed to obtain a sharp focus. At the start of each set of photographs, the patient was asked to hold an 18% gray card next to his/her face, and a picture was taken for WB correction, followed by the full set of photographs.

Photographs were taken in RAW format and processed in postproduction using Adobe Lightroom^®^.

All patients signed consent form for the use of their photographs.

The included articles showed some common aspects: 10 of 19 articles^(1-10)^ suggested the use of a telelens (60-110 mm), and only 3 of them described the working distance^(5,6,8)^. Background might vary between blue, white, black, and gray tones; however, in more than half of the included articles, the blue background was cited as preferential or alternative(1-3,6-13). Most of the articles(1-3,6,7,9,10,13-15) have suggested illumination with studio lights, four suggested speedlight only (ring flash and dedicated TTL flashes^(5,11)^ or unspecified speedlight^(16)^, and two^(17,18)^ did not mention the illumination source. Only four articles mentioned the three parameters used: ISO, aperture, and shutter speed^(3-5,8)^. More than half of the included articles did not mention these parameters^(2,6,7,10,11,13-19)^. Most of the included articles cited the Frankfurt horizontal plane for the head position^(3,5,8,9-11,13,16)^ and positioning angles such as frontal, lateral, and oblique views^(2,5,6,8,9,11-16,18)^.

Table 1 shows the results of the protocol adopted in this study for photographic documentation in oculoplasty. Illustrative photographs are shown to demonstrate the result of using this protocol.

**Table 1:** Protocol adopted in this study for oculoplastic photography

Photography in oculoplastics/Paraineters	Full face	Periorbit	Orbit	Dynamic wrinkles	Ptosis	Tumors
f-number (diaphragm)	f/8	f/8	f/8	f/8	f/8-f/11	f/11
Shutter speed (in seconds)	1/125	1/125	1/125	1/125	1/125	1/125
ISO	100	100	100	100	100	200-400
White balance	Flash mode	Flash mode	Flash mode	Flash mode	Flash mode	Flash mode
Focus	Automatic	Automatic	Automatic	Automatic	Automatic	Manual
Photographer/patient distance (in meters)	3m	1m	1m	1m	1m	0,38m
Flash	Directed toward the roof in a 75° angle and with white diffuser reflector	Directed toward the roof in a 75° angle and with white diffuser reflector	Directed toward the roof in a 75° angle and with a white diffuser reflector	Directed toward the roof in a 75° angle and with a white diffuser reflector	Frontal flash directed toward the patient’s face	Directed backward toward a white or silver 30cm light reflector
Views or projections	AP in PPG/right and left oblique	AP in PPG/right and left oblique	AP in PPG/chin elevation/all versions	Depends on the region to be treated, usually frontalis/ procerus/corrugator/ orbicularis	AP in PPG/supraversion and infraversion/with and without phenylephrine 10%	Macrophotography of the lesion/ zoomed out photography for topographic localization

* PPO= primary position of gaze;

* D= right;

* E= left; AP= anteroposterior.

**Figure 3:**
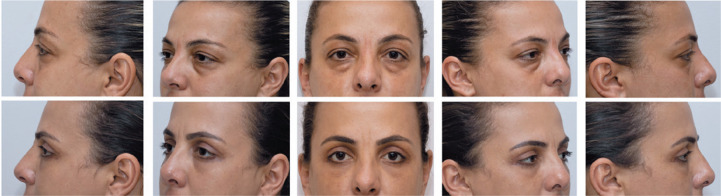
Eyelid photography. The upper photographs demonstrate the preoperative state of a blepharoplasty, primary position of gaze, right and left oblique, and lateral views. Below, the same views in the third postoperative month were taken to demonstrate background, patient positioning, framing, and lightning standardization.

**Figure 4:**
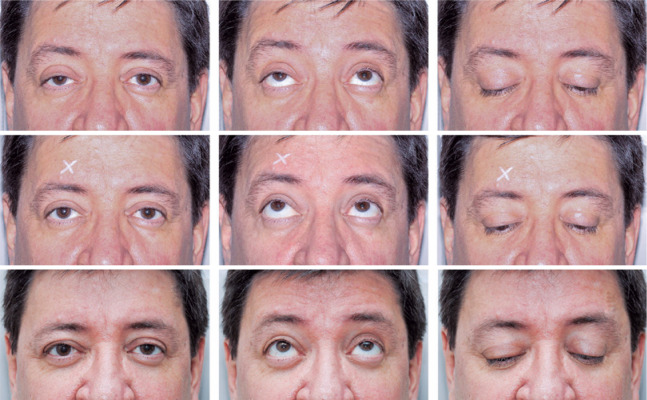
Ptosis photography. This was performed with a frontal flash to demonstrate the margin reflex distance. The first row shows the preoperative state from left to right: primary position of gaze, supra, and infraversion. The second row shows the same views, after instilling one drop of 10% phenylephrine into the right eye, as marked with an “X” above the ipsilateral eyebrow. The third row corresponds to the same views in the sixth postoperative month after a right levator aponeurosis reinsertion with a posterior approach.

**Figure 5:**
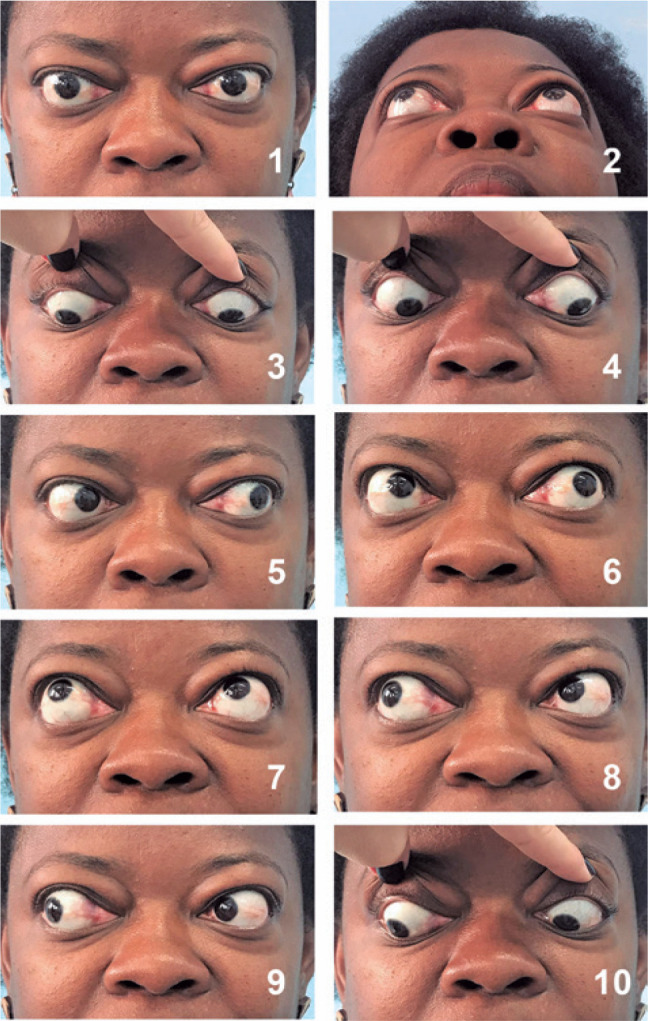
Orbit photography. These photographs demonstrate the views for orbital pathologies. In the upper left, a primary position of gaze (1), and in the upper right, the chin was lifted to demonstrate proptosis. (2) Other photographs demonstrate ocular motility in infraversion (3), infralevoversion (4), levoversion (5), supralevoversion (6), supraversion (7), supradextroversion (8), dextroversion (9), and infradextroversion (10).

We considered special angles of view and some particularities in oculoplastic photography. In pre-and post-blepharoplasty or eyelid surgery photographs, we took photographs in the PPG, right and left oblique, and lateral views (Figure 3). In cases of ptosis, we photographed the patient in PPG, supraversion, and infraversion. We also used the frontal flash to demonstrate the margin reflex distance, as this reference is the main comparison parameter for the position of the upper eyelid in the postoperative period (Figure 4). We recorded the results of the 10% phenylephrine test^(20)^, when performed, by placing a mark (white eye pencil or piece of white micropore) above the ipsilateral eyebrow where the drop was instilled. This way, when reviewing the photographs, it was clear which is the before photograph and the after-the-test photograph. In orbit photography, we recorded all gazes for the assessment and documentation of motility/restrictions of extraocular muscles. In addition, to demonstrate the anterior projection of the eyeball and proptosis, we took photographs with the camera tilted up and located below the patient’s chin, and we asked the patient to raise the chin (Figure 5).

**Figure 6:**
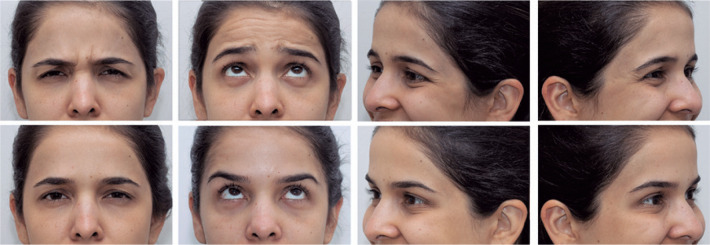
Photography in dynamic wrinkles of the face. This figure illustrates the pre- (first horizontal row) and post- (second horizontal row) state of botulinum toxin treatment. From the left to the right of the reader, the treatment of procerus and corrugators is demonstrated, patient makes an “angry face”; treatment of the frontal muscle, the patient “raised her forehead”; treatment of the orbicularis muscle (periorbital wrinkles), the patient smiled and showed the right and left oblique, respectively.

In the treatment of dynamic wrinkles and expression lines, we asked the patient to contract the facial muscle groups to be treated, as shown in Figure 6, in the preand post-botulinum toxin treatment.

**Figure 7:**
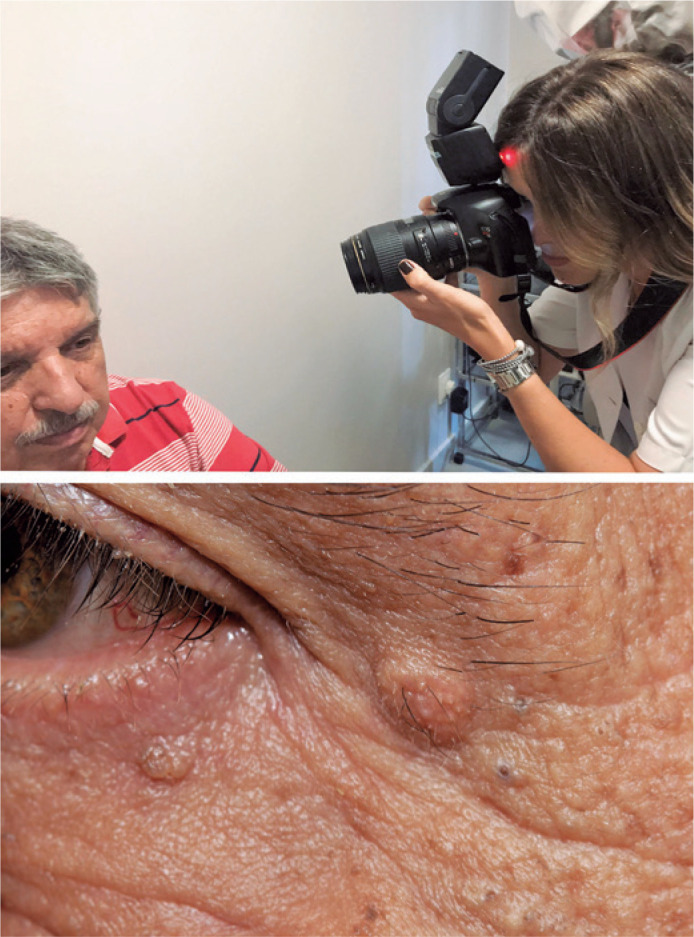
Macrophotography. These images illustrate how one can take photographs of small lesions in the periocular region using the same flash (without the need for another flash suitable for macrophotography). Thus, the focus should be placed on the manual, with a focal plane of 0.38 m, a magnification ratio of 1:1, f/11 aperture, shutter speed of 1/200, ISO of 200-400 and flash in the TTL mode directed backward with light reaching in this case, a 30-cm silver reflector. Note from the photograph of the lesion, it was possible to obtain details of the lesion without losing the portrayal of three-dimensionality since the photo was taken with bounced light.

We took macrophotographs with an external flash bent backward and bounced on a sheet of polystyrene or a white or silver diffuser reflector, as shown in figure 7.

By using the protocol described in this article, we collected data and photographs from 324 patients and obtained consistent results with optimal standardization of facial photographs before and after the surgical/cosmetic procedures.

There are three types of photographers: amateurs, professionals, and functional^(20)^. Physicians who practice oculoplastic photography fall into the category of “functional photographers,” i.e., those who are not professional photographers but need to have the minimum basic knowledge of photographic recording for their medical practice. Photodocumentation is valuable for various purposes, such as medical record keeping, insurance and legal situations, creation of models for preoperative planning and clarifying it to the patient, assessment for self-improvement, and medical education, including teaching residents, sharing data with colleagues, and preparing presentations and publications^(1-3,5,6,19-31)^.

Notably, even though the 19 articles included proposed standardization of face photographs, only two^(5,8)^ reported information of all the characteristics analyzed.

Guided by the literature review and a self-developed standardization protocol used by one of the authors (Barbi, JSF), we proposed a protocol for oculoplastic photodocumentation, which focuses on basic technical knowledge on photography, camera parameters, illumination, background, head position, and image size.

## Camera choice

DSLR cameras were recommended in several articles^(1-3,5,6,19-31)^ for their excellent cost-benefit ratio. They can offer the benefits of interchangeable lenses; thus, one can select the appropriate focal length, have complete control over camera settings, and obtain good image quality. Full-frame (24 mm x 35 mm) sensors are larger than APS-C sensors and can produce better image quality and less digital noise. However, full-frame cameras are generally more expensive, and APS-C sensor cameras are good enough for clinical photography.

## Lens

**Figure 8:**
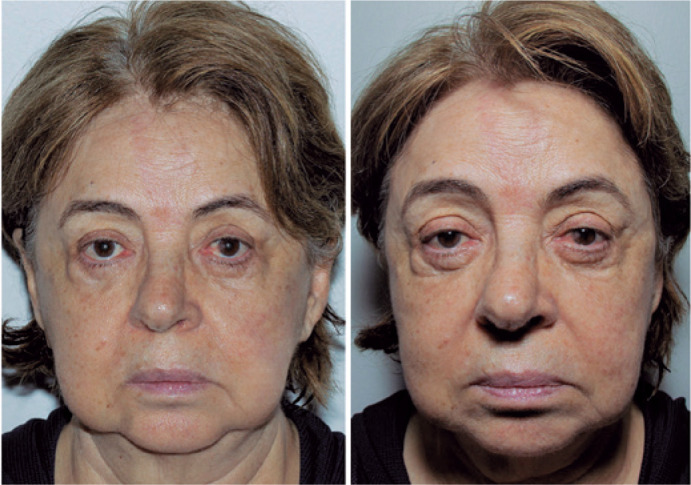
These two photographs reproduce a common error when a smaller frame of the patient’s face is desired (“magnification”). In the left photo, a 100-mm lens at 3 m from the patient was used. As it is a telephoto lens, the lens allows for the enlargement of the image and a smaller field of view, that is, the same as distancing and using the “zoom” of smartphones and compact cameras. In the right photo, we place a 35-mm lens (“normal” lens for a camera with an APS-C sensor) and brought the camera close to the patient’s face to try the same framing as that of the previous photo. Note how the second photo creates distortions on the patient’s face, with enlargement of the middle third of the face, mainly the base of the nose, an effect that can be called “Christmas ball” because it resembles the view that one can have of the face when looking at a mirrored Christmas ball.

The focal length is quoted in millimeters. With an APS-C sensor camera, lenses are regarded as “wide angle” when the focal length is <35 mm. This type of lenses delivers a wider field of view and is ideal for panoramic and landscape photography. The so-called “normal” lenses provide a viewing angle similar to that of the human eye, which corresponds to 35-mm lenses in cameras with APS-C sensor. Telephoto lenses provide a smaller viewing angle and magnification of the image and are ideal for face and close-up photographs^(5,21,24,26)^. Such lenses avoid the face distortions that can occur with the approach of normal or wide-angle lenses (Figure 8)^(10,16)^. The lenses can be fixed focal length (prime lenses) or zoom lenses with adjustable focal length. Fixed lenses are those with a single focal length, and to change the frame, the photographer must move back and forth. Given the same objective, zoom lenses allow several focal lengths that can be adjusted manually, there is no need for the photographer to move just to change the frame.

In medical practice, fixed lenses are recommended for two reasons: they allow better image quality and easier standardization. The authors of this article recommend a 100-mm macro lens, according to personal experience and published articles^(1-3,6-10,25,26)^. The authors recommend using this type of lenses because they allow greater distance between the photographer and the patient, which is favorable for lighting, as it bounces back, has a longer path to reach the patient, and delivers a softer and broader illumination. A dedicated macro lens is preferred, as it allows closer focusing on a 1:1 magnification ratio of the patient^(5)^. This feature is advantageous for photographing small lesions at large magnification.

## ISO, aperture, and shutter speed

Cameras interact with light by basically three components in the camera settings, the so-called light photographic triangle: diaphragm or aperture (f-number), shutter speed, and ISO^(23-25)^. In an easy-to-understand and didactic way, we can compare these parameters with the physiology of the eye. The diaphragm of a camera can be compared to the pupil; with a larger aperture, more light enters and the shallower the depth of field and vice versa. The shutter speed could be compared with the act of “blinking” It works as a window that allows light to enter the eye depending on how long it is open or how fast it closes. The ISO is a measure of the sensitivity of the sensor to light, equivalent to the retina, and can be adjusted according to environmental brightness. In a very bright environment, the ISO should be reduced and vice versa.

These three parameters can be adjusted automatically, semi-automatically, or manually on DSLR cameras. For correct standardization, the photographer should always choose the manual mode and determine these values for consistency in the photographs before and after surgical or cosmetic treatments.

A camera’s aperture is quantified by the “f” number, which is a ratio of the lens focal length and the diameter of the aperture. The aperture of the diaphragm in a 100-mm f/2.8 macro lens ranges from 2.8 to 22; the smaller the value, the more open the diaphragm will be and the smaller the depth of field. In medical practice, it is recommended to work with apertures between f/5.6 and f/8 for two reasons: (1) to have sufficient depth of field to make the whole face of the patient sharp (very wide diaphragms can leave the eyes focused and the tip of the nose and ears blurred), and (2) in a 100-mm macro lens, these “f” values allow for better image quality (in very open or very closed diaphragms, there is a slight loss in image quality).

The shutter speed is a measure of how long the camera’s shutter blades are open to expose the sensor to the light, and it is measured in seconds or fractions of a second. In the present study, the shutter speed of the camera ranges from 30 s (slowest) to 1/4000 seconds (fastest). When the flash is attached to the camera shoe, this command is limited to a maximum speed of 1/125 (corresponding to the maximum synchronization speed of the flash). At very high speeds, the second shutter curtain may close before the light hits the entire sensor, which generates a dark band on the photographs. To prevent this, the camera limits fast shutter speeds when the flash is attached. Since the ambient light in an ophthalmology outpatient clinic is not consistent in brightness and color temperature^(5)^, the maximum flash sync speed should be used to exclude ambient light interference in the photographs. Thus, regardless of the time of the day, sunlight in windows, and ceiling lights (on or off), the photograph will have light consistency because the camera will record the light coming only from the flash.

For the same reason mentioned above, the photographer should work with lower ISO values, and 100 is suggested. The ISO represents the sensitivity of the sensor to light, and lower values are desired so that there is minimal or no capture of ambient light.

## Illumination

Although most articles encourage the use of studio lights^(1-3,6,7,9,10,13-15)^, the authors suggest using a unique light source (TTL speedlight) (1) to simplify the photography equipment as studio lights may not be viable in terms of space in ophthalmology offices and 2) because a speedlight works in very effectively without requiring additional lights for full face and periorbital region photographs.

In addition to the photographic triangle, the power level of the flash is the fourth element during flashed photographs. This parameter is set on the flash itself and can be placed in manual or automatic mode. This study agrees with Ong et al.^(5)^, who suggested the use of the flash in automatic mode, that is, the camera will determine the power of the flash using the TTL metering function. In a TTL system, the camera fires a quick pre-flash to determine the amount of light needed to illuminate the subject followed by the correct flash output to achieve correct exposure. If photographs are always taken at the same working distance using the same lens, the photographs will have good consistency in this parameter even when working with the flash in automatic mode. However, as most of the oculoplastic surgeons are not using a studio, factors, such as furniture or patient clothing color, may cause small changes in automatic reading. To avoid handling the flash in manual mode, the authors suggest using an 18% gray card in the first photo in a series. The card helps with the WB in the editing software in postproduction, as it serves as a neutral color reference for adjustment of the color temperature of the photographs^(23,24)^.

**Figure 9:**
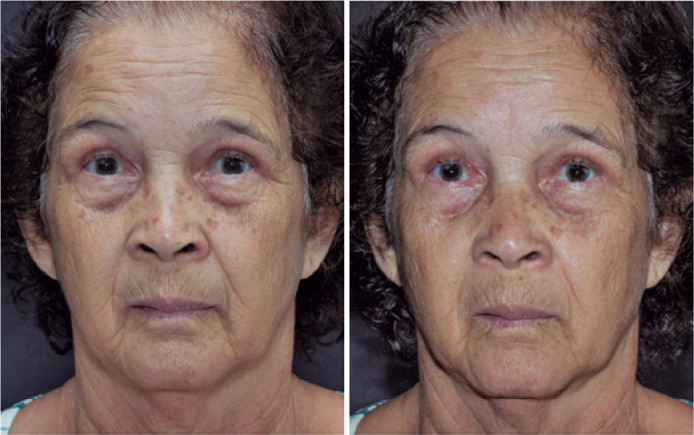
A common error when using the flash. The left photograph was taken with an external flash bounced on the ceiling, and the right photo was taken with an external flash directed straight at the patient’s face (frontal flash). Note how relief (such as the apparent fat pockets of the lower eyelids), shadows (such as the nasojugal groove), and wrinkles and skin marks are more clearly shown in the first photograph. In the second photograph, the frontal flash “flattens” the image, removing the idea of three-dimensionality and favoring the patient in the sense of disguising skin wrinkles and pigmentations, which is not suitable for medical photographs in which one wishes to show the face with all its natural features.

Another important suggestion is the use of the flash directed toward the ceiling, thus using the bounced light to achieve smoother and broader lighting and greater idea of three-dimensionality. The frontal flash results in harsh light, which leaves the photo “flat”, that is, without any portrayal of relief and contours that are fundamental for medical photodocumentation^(32)^ (Figure 9). In addition, a white diffuser reflector attached to the flash is also recommended to direct part of the light in a straight line, which will reach the patient’s face much more smoothly, as it is bounced, not direct light. This reflector is used to eliminate shadows that can occur in the periorbital region when the lighting is exclusively done from “top to bottom” when using only light reflection from the ceiling. This is useful in men who have a very prominent forehead. These “shadows” in the periocular region can be eliminated or smoothened. This this problem can be also solved by the use of a small rectangular reflecting panel positioned horizontally against the patient’s chest, just under the collarbone, outside the framing, just to reflect the light from bottom to top, minimizing these shadows^(25)^. For macrophotographs, the current literature^(33-36)^ suggests the use of a ring or twin flash, specific for this type of photographs. However, as this study aimed to simplify the photographic apparatus without loss of quality and consistency of images, the authors used the external flash aimed at a white reflector (which can be something as simple as a white card) or a 30-cm silver reflector located behind the photographer. We chose this method because the photographer-patient distance for macrophotography is approximately 0.38 m, which is very close. The use of the front flash will cause the flattening of lesions, removing the same important characteristics as shadows, and raised edges. Reflecting the light in the ceiling is not also a good option, as in macrophotography, the area framed reflecting light is very small and needs greater illumination than the light reflected from the ceiling, leaving the image dark. Directing the flash backward into a reflector allows the light to smoothly illuminate the lesions, without erasing its contour and relief, which should be demonstrated in the preoperative record or clinical follow-up.

## WB

WB is a process of removing the unnatural color cast from an image^(1,7,14,32,34,35,37,38)^. Different light sources can have different color temperatures. Fluorescent lamps can result in greenish images, just as incandescent sources can result in orange skin tones^(23-25)^. In medical photography using the proposed protocol, since there will always be a flash, it is recommended to leave the WB in “flash” mode. As previously said, if the flash perhaps “misses” the light reading of the scene, the WB can be corrected by using an 18% gray card in the editing software.

## Background

The need for a uniform background is well established, but the color suggested for this background varies between authors. Several articles of medical photography^(1-3,6,8-10,12,26)^ have suggested the use of a blue background because it is opposed to the yellow shade of the skin of most patients, generating a composition pleasant to the human eye. Some authors^(5)^ suggest a black background to eliminate shadows that could be generated by a white background, depending on the incident light. However, a black background may present challenges in patients with dark hair and cannot provide subjectbackground separation unless another source of light is used, making office photography more complex^(7)^. The simplest and cheapest way is to paint the wall with a color close to 18% gray based on established photography books^(23,24)^. This tone is intermediate between white and absolute black, and it helps with the photometer readings built in DSLR cameras. In addition, due to its “absence of color”, it prevents unwanted reflections of color by the flash on the patient’s face. The suggestion was to paint the office ceiling white, as it will be used to bounce the light from the dedicated flash. Moreover, matte paint should be used to avoid over-reflection of the light^(27)^.

## Head position

The recommended positions for full-face photographs are anteroposterior, right anterior oblique, left anterior oblique, right profile, and left profile(5,6,8,9,11-16,18,26,28,30).

Discrete marks on the floor are also essential to determine the distance between the photographer and the patient^(30)^. The use of the thirds grid on the camera’s screen is highly recommended to help the photographer align the camera precisely^(6,15,26)^.

The authors agree with the statements of Rhee^(19)^ regarding a frame reference, i.e., a horizontal line that passes through the center of the pupil or eyelid canthus and the apex of the ear, because this reference can be used in frontal, oblique, and profile pictures. More than half of the included articles used Frankfurt’s horizontal plane (a line that passes through the external auditory canal and the inferior orbital rim) as a reference for head positioning^(3,5,8-11,13,16)^. Nevertheless, it is a radiological reference, and it can be difficult to reproduce in photography.

To frame the face in the oblique position, the patient rotated his/her entire body, facing markings on the floor corresponding to 45**°** on the right and then on the left^(11,28)^. These markings correspond to the patient rotating his/her body until the patient’s tip of the nose aligns with the malar eminence and the ear apex with the lateral eyelid canthus. For lateral views, the ear apex should be aligned to the lateral eyelid canthus and only the ipsilateral Cupid’s bow should be seen.

Although photographs of the face are recommended in a vertical orientation^(29,30)^, the authors recommend the horizontal orientation for the ease of operation with the camera and flash.

In orbital diseases, the apparent exophthalmos and enophthalmos must be documented. Thus, the authors recommend the “worm view” which is taken with the patient in neck extension and the camera viewing from below; the tip of the nose aligns with the glabella, right in the middle of the eyebrows, the focus is kept in the eyes, and both eyes appear in the frame.

## Image size

Regarding the format of the photographs, the suggestion was to shoot in RAW format^(1,21)^, which is a type of file without processing in which color information, WB, and other parameters will be edited later in postproduction using the Adobe Lightroom® for editing, converting RAW to JPEG files (without loss of quality since the files will not be compressed), and storing them in the cloud, which allows for device synchronization and therefore easy sharing or exchange of files.

A narrative review on photodocumentation in facial surgery was performed, and a self-developed, guided by the literature review, standardized protocol for facial photographic registration was described. Preand postphotographs of surgical and cosmetic procedures were collected from 324 patients using this protocol, and consistent results were obtained with optimal standardization of facial photographs. This protocol can be easily adapted to any oculoplastic surgeon’s practice, without the need to set up a studio in the office or spend on unnecessary extra photographic equipment.

Essentially, there is not a gold standard protocol in facial photodocumentation. However, there is agreement on the importance of standardization and reduction in variables as much as possible to achieve consistency across photographs and register the patient’s condition and clinical evolution as accurately as possible.
